# Risk Assessment in Secondary Mitral Regurgitation: A Step Toward Clarity?∗

**DOI:** 10.1016/j.jacadv.2022.100074

**Published:** 2022-08-26

**Authors:** Wendy Tsang, Rashmi Nedadur

**Affiliations:** a Division of Cardiology, Toronto General Hospital, University Health Network, University of Toronto, Toronto, Canada; b Division of Cardiovascular Surgery, University of Toronto, Toronto, Canada

**Keywords:** artificial intelligence, echocardiography, machine learning, mortality, outcomes, secondary mitral regurgitation

**Figure:**
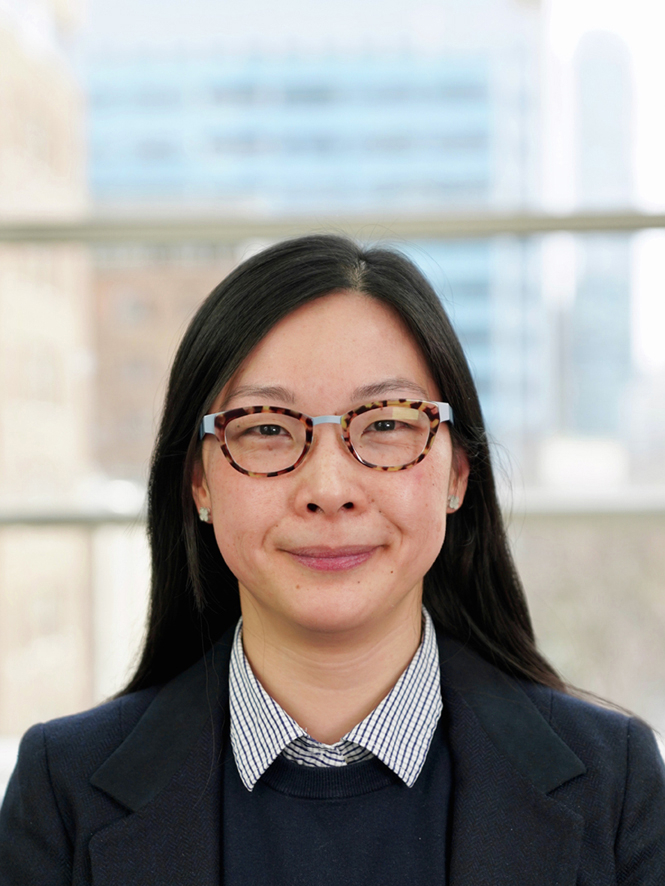

It is well established that secondary mitral regurgitation (sMR) is associated with poor outcomes regardless of left ventricular (LV) function and that mitral valve interventions could improve outcomes in this population.^1^ However, identification of those who would benefit most from intervention is unclear. Recent percutaneous mitral edge-to-edge repair trials have indicated that echocardiographic parameters related to LV dimensions and function could offer guidance in decision-making.^2^, ^3^, ^4^, ^5^, ^6^ But, by focusing only on these echocardiographic parameters, we ignore the wealth of clinical and laboratory data available that could improve patient selection. Historically, performance of studies that could include these data have not been executed because of limitations in traditional statistical methods. Developments in machine learning have now provided the tools that could be applied to this problem.^7^

In this issue of *JACC: Advances*, Heitzinger et al^8^ apply machine learning methods to develop a structured decision tree–like approach to risk stratify patients with severe sMR and a wide range of LV function. The authors used contemporaneously collected clinical, echocardiographic, and laboratory variables from 1,317 severe sMR patients. Their primary outcome was all-cause mortality. First, using 70% of the entire cohort, the authors performed univariate Cox proportional hazards ratio analysis followed by bootstrap resampling on only the clinical, the laboratory, or the echocardiographic variables to identify parameters associated with mortality for inclusion in a type of supervised learning called survival tree modeling. Then, they performed survival tree-based modeling with analysis stopped when there were 95 patients left in the terminal leaves. Finally, parameter cutoffs determined from this analysis were applied to the remaining 30% of their sMR cohort for validation using Kaplan-Meier and univariate Cox proportional hazards regression. The authors then repeated each step to identify parameters and their cutoff for the 3 heart failure (HF) subtypes within their sMR population. These subtypes included HF with preserved ejection fraction (left ventricular ejection fraction (left ventricular ejection fraction [LVEF]) >50%), HF with mildly reduced ejection fraction (LVEF 40%-50%), and HF with reduced ejection fraction (LVEF <40%). Because of the small numbers within each HF subgroup, there was no division into derivation and validation subgroups for this analysis.

From the entire sMR cohort, after the first step, age and peripheral artery disease were most associated with all-cause mortality in the clinical variables analysis, LV end-diastolic diameter and the presence of mild, moderate, or severe LV dysfunction in the echocardiographic analysis, and blood urea nitrogen (BUN), creatinine, and albumin in the laboratory analysis. These parameters were then used in the survival tree modeling, and 8 subgroups were identified that differ in their long-term survival. A near 20-fold risk difference in mortality was seen between the highest and lowest risk subgroups. The lowest risk subgroup had survival of 97% and 85% at 1 and 6 years, respectively, and the highest risk subgroup had survival of 48% and 11%. Patients with low risk of death were younger patients (aged <66 years) with normal hemoglobin (>12.7 g/dL) and albumin (>40.6 g/L) levels. Patients with higher risk of death were older (aged >68 years) with reduced albumin (<40.6 g/L) and significantly elevated BNP (>9,570 pg/mL) levels. When these parameter cutoffs were applied to the validation cohort, there was differentiation into similar survival.

The authors then repeated their analysis on each of the 3 HF subtypes within their sMR population. They found that in HF with preserved ejection fraction, age, female sex, bilirubin, BUN, cholesterol, and albumin were the most important parameters associated with all-cause mortality. The use of these parameters in survival modeling identified 3 survival subgroups with the poorest survival associated with elevated BUN. For HF with mildly reduced ejection fraction, atrial fibrillation, body mass index, LV end-diastolic diameter, albumin, and aspartate transaminase were most associated with survival, and when entered into the survival model, those with reduced albumin had the worst survival. Finally, for the HF with reduced ejection fraction, age, peripheral arterial disease, LV end-diastolic diameter, and N-terminal pro-brain natriuretic peptide (NT-proBNP) were the most important parameters, with NT-proBNP playing a significant role in the survival model.

This study has several unique strengths and findings. First, by using commonly collected variables and studying a wide range of sMR patients, the authors increased the generalizability of the developed risk stratification algorithms for routine clinical practice. This study also illustrated that sMR represents a heterogeneous population and that a semi-tailored approach may be needed for both assessment and management. LVEF could triage patients to different algorithms that require distinctive clinical, echocardiographic, or laboratory parameters for risk assessment. Although this semi-tailored approach may create more complexity during assessment, it could potentially lead to improved management pathways by refining how the sMR population is grouped and studied, potentially leading to better medical therapies and interventional recommendations. Finally, the parameters identified by this study (NT-proBNP, LV end-diastolic diameter, renal failure, hypoalbuminemia) are consistent with prior publications examining mortality risk in mitral regurgitation patients.^9^^,^^10^

This study also demonstrates the value of machine learning methodology in expanding our understanding of sMR pathophysiological pathways and patient trajectories in an unbiased, data-driven way. Previous work, also by this group, used a similar clustering analysis after principle component dimensionality reduction analysis to identify high-risk sMR groups but focused on left atrial anatomical indexes in keeping with the atrial functional sMR concepts published by Reddy et al^6^^,^^11^ In this study, Heitzinger et al^8^ further that work by including clinical and laboratory markers in addition to echocardiographic measurements for risk stratification.

One of the most intriguing findings in this study is that echocardiographic measurements did not appear to contribute to the 8 identified mortality-related strata from examining the overall sMR cohort. Measures of general clinical status and end-organ damage were far more predictive of all-cause patient mortality. Whether a different result could have been obtained if cardiovascular mortality was the primary outcome is unclear. The lack of echocardiographic parameter significance for all-cause mortality could also be related to the general advanced stage of these patients with severe sMR, and similar to other studies, including patients with moderate or greater sMR could have perhaps identified echocardiographic measures.^6^ The lack of echocardiographic core laboratory analysis and the use of semiqualitative assessment for parameters such as right ventricular function and tricuspid regurgitation severity could have also contributed to these results. It is interesting that left atrial dimensions and tricuspid regurgitation severity did not differ among the HF subtypes. The use of left atrial volume, as opposed to left atrial diameter, could have been a more sensitive parameter for inclusion in the modeling. Similarly, the impact of LV end-diastolic and end-systolic volumes and strain and right ventricular area in the modeling would have been informative, especially given recent interest in the relationship between mitral regurgitant volume and LV volumes.^12^ Finally, measures of importance in this stratification, such as age, renal, and liver function, may be collinear to the changes in the echocardiographic measures observed in sMR, therefore rendering them unimportant compared with these clinical and laboratory markers. This finding could also highlight some of the limitations on the use of echocardiography once severe sMR is present.^6^ Perhaps, tests such as cardiac magnetic resonance imaging could be used in these circumstances by providing additional information on myocardial fibrosis.

Other limitations to this study include the inclusion of patients who had undergone surgical or percutaneous mitral valve intervention (n = 200), cardiac transplantation (n = 29) or received an LV assist device (n = 1) or implantable cardiac defibrillator (n = not reported). Although the patients who underwent mitral valve intervention mainly had normal LV function compared with those receiving cardiac transplantation, the outcome trajectory of all these patients presumably would have been changed by their procedures, and so a sensitivity analysis excluding these patients would have been useful, especially in the HF subgroup analysis. In addition, it would be useful to compare the survival tree generated with and without feature selection, as the feature selection process reduces the granularity of the complex data used in model training. Finally, the HF subgroup analysis would benefit from validation.

In summary, Heitzinger et al^8^ have furthered our understanding of mortality risk in severe sMR patients. It is clear that severe sMR patients are not a homogenous population whose risk profile can be easily defined. Instead, stratification requires understanding the complex interplay of many factors. In addition to LV size and function, mitral regurgitation severity, and left atrial size and function, we can now appreciate that comorbidities and general health status play a significant role in the clinical outcomes of patients with severe sMR. These studies could be expanded even further to include patient symptom status for an even more nuanced identification of patients that could benefit from intervention. Ultimately machine learning with multimodality integration with clinical and laboratory data, imaging, and electrophysiology could allow precision medicine for the identification of sMR patients who could truly benefit from mitral valve intervention (Figure 1).^13^

**Figure 1:**
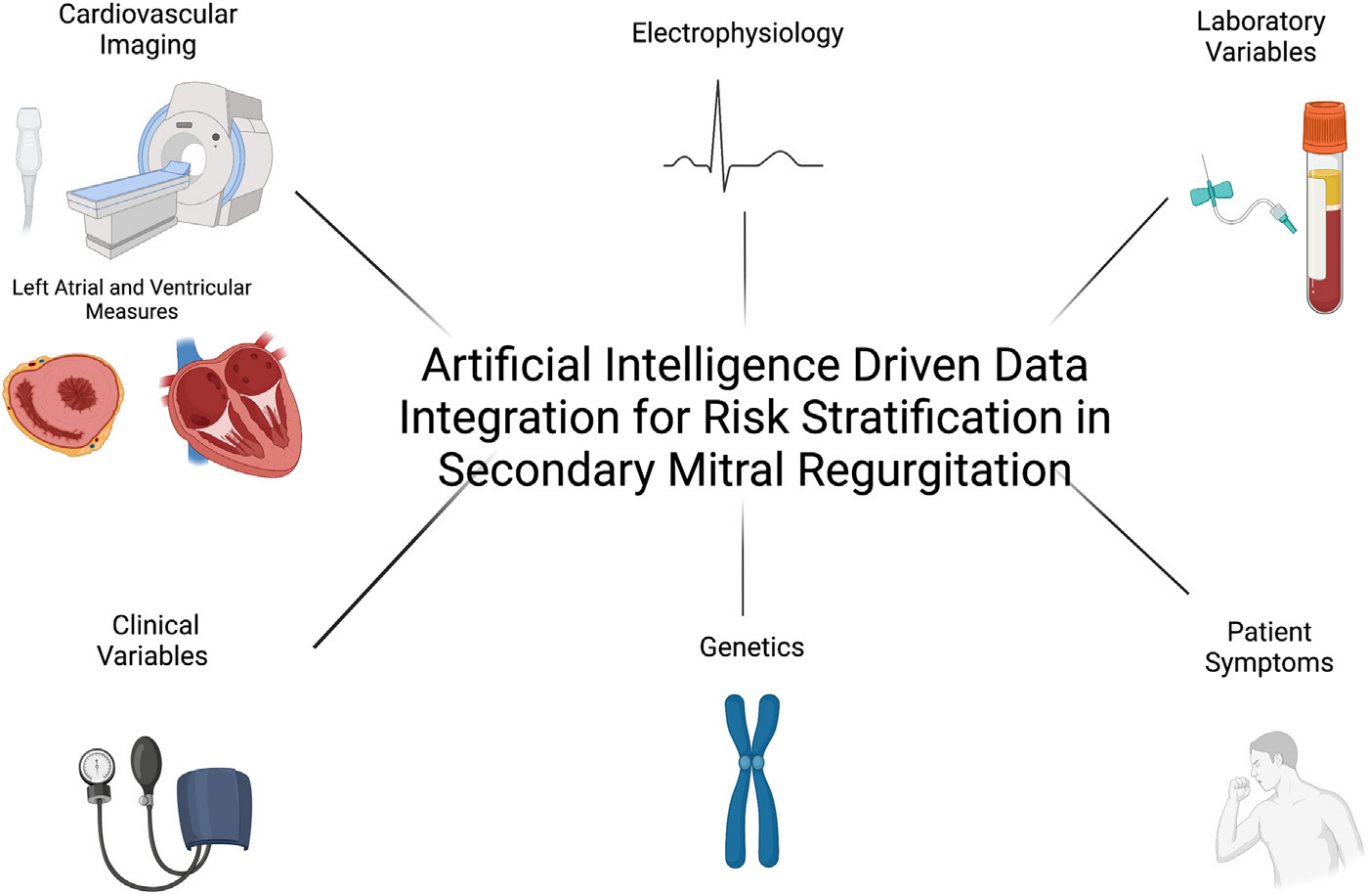
Future of Secondary Mitral Regurgitation Risk Stratification Secondary mitral regurgitation risk stratification in the future could use artificial intelligence to integrate multimodal data to identify patients who would benefit from medical therapy or intervention and so, optimize the outcomes of this high-risk population. Created with BioRender.com.

## Funding support and author disclosures

Dr Tsang is supported by a Heart and Stroke Foundation of Canada National New Investigator Award; and has received research support from the Canadian Cardiovascular Society, Philips Healthcare, and Siemens. Dr Nedadur has reported that he has no relationships relevant to the contents of this paper to disclose.
